# A curated transcriptome dataset collection to investigate the functional programming of human hematopoietic cells in early life

**DOI:** 10.12688/f1000research.8375.1

**Published:** 2016-03-30

**Authors:** Mahbuba Rahman, Sabri Boughorbel, Scott Presnell, Charlie Quinn, Chiara Cugno, Damien Chaussabel, Nico Marr

**Affiliations:** 1 Sidra Medical and Research Center, Doha, Qatar; 2 Benaroya Research Institute, Seattle, WA, USA

**Keywords:** transcriptomics, fetal, peripheral blood, umbilical cord blood, immune ontogeny, hematopoietic cells, PBMC, T cells, Tregs, B cells;

## Abstract

Compendia of large-scale datasets made available in public repositories provide an opportunity to identify and fill gaps in biomedical knowledge. But first, these data need to be made readily accessible to research investigators for interpretation. Here we make available a collection of transcriptome datasets to investigate the functional programming of human hematopoietic cells in early life. Thirty two datasets were retrieved from the NCBI Gene Expression Omnibus (GEO) and loaded in a custom web application called the Gene Expression Browser (GXB), which was designed for interactive query and visualization of integrated large-scale data. Quality control checks were performed. Multiple sample groupings and gene rank lists were created allowing users to reveal age-related differences in transcriptome profiles, changes in the gene expression of neonatal hematopoietic cells to a variety of immune stimulators and modulators, as well as during cell differentiation. Available demographic, clinical, and cell phenotypic information can be overlaid with the gene expression data and used to sort samples. Web links to customized graphical views can be generated and subsequently inserted in manuscripts to report novel findings. GXB also enables browsing of a single gene across projects, thereby providing new perspectives on age- and developmental stage-specific expression of a given gene across the human hematopoietic system. This dataset collection is available at: http://developmentalimmunology.gxbsidra.org/dm3/geneBrowser/list.

## Introduction

Human immune defenses are highly dynamic and vary with age, reflecting the different environmental challenges and needs for adaptation during the fetal, neonatal and postnatal period, and throughout life. Not surprisingly, functional differences of the human immune system are most profound very early in life due to the limited antigen exposure *in utero*, and a variety of developmental, maternal, nutritional, and environmental factors that can act in concert to modulate innate and adaptive effector functions of hematopoietic cells ^1– 3^. At the same time, newborns and young infants are particularly vulnerable to infection, with each developmental stage representing a ‘window of vulnerability’ to a very specific subset of pathogenic microbes ^4^. In this context, an increasing number of studies have been designed to gain a deeper understanding of immunity in early life, and ultimately, to reveal the underlying immune defense and regulatory mechanisms that determine the clinical outcome of primary infections, and responses to early childhood vaccination ^1– 3^. Nonetheless, our understanding of the developing immune system in early life remains very limited, in part because of the difficulty to access biological specimen from human fetuses, neonates, and young children. Most often, *in vitro* studies utilizing umbilical cord and peripheral blood samples were used to assess neonatal immune defenses, and in particular to reveal critical differences in the functional programming of neonatal hematopoietic cells in comparison to that of adults. Aside from the limited repertoire of memory B and T lymphocytes in neonates, such studies have revealed substantial gestational- and postnatal age-dependent differences in the phenotype and function of a variety of hematopoietic cell types upon *in vitro* stimulation of (whole) cord/peripheral blood and isolated blood mononuclear cells with a variety of immune stimulators and modulators, including purified Toll-like receptor (TLR) and RIG-I-like receptor (RLR) agonists, cytokines, and whole pathogens, which engage a variety of pattern recognition receptors (PRRs) and signaling pathways ^5– 14^. The underlying reasons for the functional differences between hematopoietic cells obtained at different gestational and postnatal ages remain largely unclear. There is little evidence for postnatal age-specific variation in the PRR gene expression at baseline (i.e. in the absence of infection or *in vitro* stimulation) ^8, 15, 16^, suggesting critical differences in downstream signaling networks and regulatory mechanisms by which hematopoietic cells exert their specific effector functions. These age-specific differences have yet to be revealed.

Here we make available, via an interactive web application, a curated collection of transcriptome datasets of either whole blood samples, isolated blood mononuclear cells, or a variety of sort-purified hematopoietic cell populations obtained from human neonates or fetal tissue. In the selected datasets, transcriptional profiles were obtained in the absence or presence of various intrinsic and exogenous immune modulators. For comparison, these datasets contain samples from other age groups (most often from healthy adult volunteers), or cell populations at multiple differentiation stages. The ability to pool and analyze samples across various age and risk groups, and across various hematopoietic cell types, offers a unique opportunity to define common denominators of early life immunity and to reveal critical differences in the functional programming of fetal and neonatal hematopoietic cells.

To this date, over 65,000 high-throughput functional genomics studies have been deposited in the NCBI Gene Expression Omnibus (GEO), a public repository of transcriptome profiles. However, identifying datasets relevant to a particular research area is not straightforward, because GEO is primarily designed as a repository for the storage of data, rather than browsing and interaction with the deposited data. Thus, we used a custom interactive web application, called the Gene Expression Browser (GXB) ^17^, to host the datasets we identified as particularly relevant to reveal gestational and postnatal age-specific differences in the gene expression pattern of fetal and neonatal hematopoietic cells. GXB allows seamless browsing and interactive visualization of our GEO dataset collection containing large volumes of heterogeneous data, such as transcriptome profiles, demographic information, as well as clinical information. Users can easily customize data plots by adding multiple layers of information (such as postnatal age, weeks of gestation at birth, and gender), modify the ordering of samples and genes, change the plot type, and generate links (mini URLs) capturing the user’s settings, which can then be inserted in email communications or in publications. These user-generated mini URLs provide access not only the transcription data but also to rich contextual information and data interpretation, including gene information, relevant literature, a description of the study design, as well as detailed sample information that was supplied along with the transcriptome data submission to GEO.

## Material and methods

**Figure 1.**
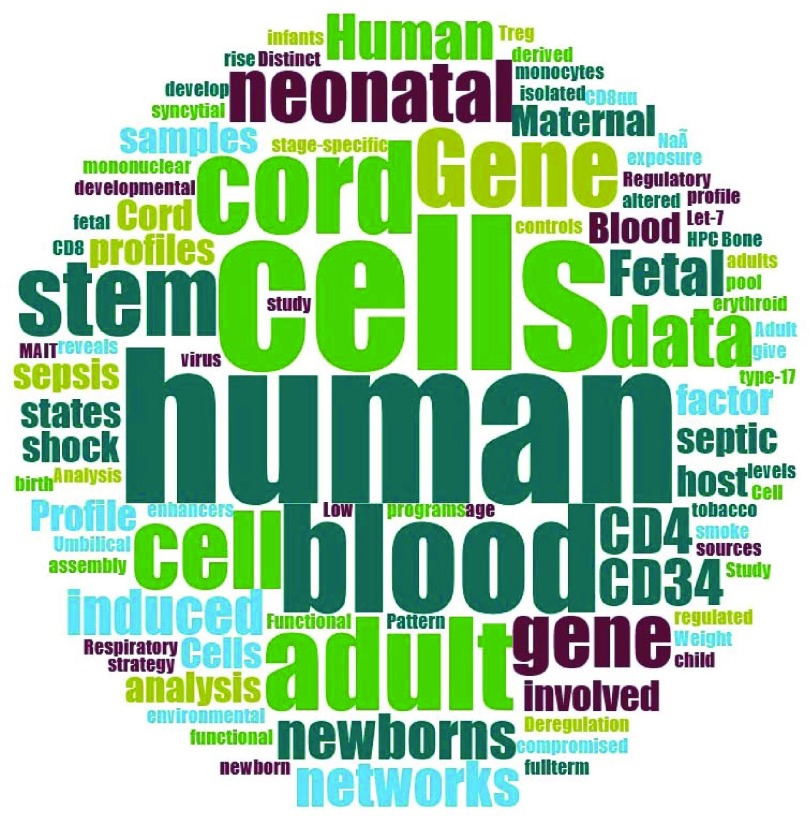
Word cloud generated from the title of published journal articles where the datasets were first reported, or the dataset title if no journal article was available. The word size is proportional to the frequency of each word.

Potentially relevant datasets deposited in GEO were identified using two search queries which were designed to retrieve entries where the title and description of the datasets contained the words newborn OR neonate OR neonatal OR fetal OR cord. The search was restricted to datasets that were generated from human whole blood, human blood mononuclear cells, or sort-purified human hematopoietic cells using Illumina or Affymetrix platforms. Studies on cancer patients or cell lines were excluded. First, the following query was used: Homo sapiens[Organism] AND (newborn[DESC] OR neonate[DESC] OR neonatal[DESC] OR fetal[DESC] OR cord[DESC]) AND (blood[DESC] OR PBMC[DESC] OR PBMCs[DESC] OR lymphocyte[DESC] OR lymphocytes[DESC] OR "B cell"[DESC] OR "B cells"[DESC] OR "plasma cells"[DESC] OR "T cell"[DESC] OR "T cells"[DESC] OR Treg[DESC] OR Tregs[DESC] OR monocyte[DESC] OR monocytes[DESC] OR dendritic[DESC] OR DC[DESC] OR DCs[DESC] OR "natural killer"[DESC] OR NK[DESC] OR NKT[DESC] OR neutrophil[DESC] OR neutrophils[DESC] OR erythroblast[DESC] OR erythroid[DESC] OR CD19[DESC] OR CD20[DESC] OR CD3[DESC] OR CD4[DESC] OR CD8[DESC] OR CD71[DESC]) AND ("Expression profiling by array"[gdsType] OR "Expression profiling by high throughput sequencing"[gdsType]) NOT (cancer[DESC] OR leukemia[DESC] OR lymphoma[DESC] OR "cell line"[DESC] OR myeloma[DESC] OR mesenchymal[DESC] OR endothelial[DESC]). In addition, we used the following query to specifically retrieve datasets containing samples from neonatal sepsis patients: sepsis AND (neonate OR newborn). In total, more than 450 datasets were retrieved by the two queries. The list of datasets retrieved from the 2 queries was manually curated and restricted to datasets that: (i) contained transcriptional profiles from primary hematopoietic cells; (ii) contained samples of fetal or neonatal origin; (iii) contained a minimum of 3 samples (i.e. biological repeats) for each of the major variables assessed in the respective study; and (iv) allowed within the same dataset, the comparison of transcriptional profiles either between different age groups (e.g. neonate versus adult), between infants born at different gestational ages, between different risk groups (e.g. infants with low birth weight versus those with normal birth weight), or between cell differentiation stages. This process involved reading through the descriptions and examining the list of available samples and their annotations. For the filtering of the dataset list, the Bioconductor package GEOmetadb, version 1.30.0, and its SQLite database was used to capture detailed information on selected GEO datasets in a single table ( https://www.bioconductor.org/packages/release/bioc/html/GEOmetadb.html) ^18^. Sometimes, it also required accessing the original published report in which the design of the study and generation of the dataset is described in more detail. Using the stringent criteria detailed above, we reduced the list down to 41 GEO datasets (excluding SuperSeries), of which 32 GEO datasets were uploaded into our interactive web application, GXB, together with corresponding SuperSeries if available (4 additional GEO datasets). For the remaining datasets the platform used to generate the transcriptome profiles was not supported by GXB (9 datasets). Out of the 32 curated datasets, 8 include samples of fetal origin, and 25 datasets include samples of neonatal origin, usually in conjunction with samples of adult subjects (including 3 datasets containing peripheral blood samples from the mothers). The majority of neonatal samples were obtained from healthy subjects, mostly utilizing umbilical cord blood. In these studies, a variety of factors were assessed that may induce and/or reveal differences in the functional programing of neonatal hematopoietic cells, including the effect of active/passive smoking of the mothers during pregnancy ( GSE27272, GSE30032) ^19, 20^, standards of living and hygiene ( GSE53471, GSE53472, GSE53473) ^21^, as well as *in vitro* exposure of neonatal and adult cells to purified TLR ligands ( GSE67057, GSE3140), and to whole pathogens ( GSE24132). In 6 studies, peripheral blood samples were obtained from babies with neonatal sepsis ( GSE25504, GSE26440, GSE26378, GSE69686) ^22– 24^ bronchopulmonary dysplasia ( GSE32472) ^25^, or from babies with low birth weight ( GSE29807). The transcriptional profiles were either generated from whole blood (11 datasets), cord and peripheral blood mononuclear cells (1 dataset), or a variety of sort-purified hematopoietic cell populations at different differentiation stages, including cells derived from neonatal and adult hematopoietic stem cells as well as erythroid cells. The latter cells have recently been shown to play an important immunosuppressive role in the context of neonatal infection ^26^. The datasets that make up our collection are listed in Table 1. We also generated a word cloud from the title of published journal articles where the datasets were first reported (or the dataset title if no journal article was available), which provides information on the type of studies that make up our dataset collection ( Figure 1).

**Table 1.** List of curated datasets. WB, whole blood; HSC, hematopoietic stem cells; DC, dendritic cells; QC, quality control; NA, not applicable ( http://developmentalimmunology.gxbsidra.org/dm3/geneBrowser/list).

Title	Platform	Sample source		QC Markers	Number of Samples	GEO ID	Ref.
		Origin	RNA				
Whole blood mRNA expression profiling of host molecular networks in neonatal sepsis (platforms GPL6947)	Illumina	neonatal	WB	XIST	63	GSE25504	22
Whole blood mRNA expression profiling of host molecular networks in neonatal sepsis (platform GPL13667)	Affymetrix	neonatal	WB	XIST	20	GSE25504	22
Whole blood mRNA expression profiling of host molecular networks in neonatal sepsis (platform GPL570)	Affymetrix	neonatal	WB	XIST	5	GSE25504	22
Expression data for derivation of septic shock subgroups	Affymetrix	neonatal, pediatric	WB	NA	130	GSE26440	23
Expression data from validation cohort of children with septic shock	Affymetrix	neonatal, pediatric	WB	NA	103	GSE26378	23
Post-natal age is a critical determinant of the neonatal host response to sepsis	Affymetrix	neonatal	WB	NA	150	GSE69686	24
Maternal influences on the transmission of leukocyte gene expression profiles in population samples (mother and child)	Illumina	neonatal, adult	WB	NA	56	GSE21342	29
Standard of hygiene and immune adaptation in newborn infants [113 cord blood RNA samples] (This SubSeries is part of SuperSeries GSE53473: Standard of hygiene and immune adaptation in newborn infants)	Affymetrix	neonatal	WB	XIST	113	GSE53471	21
Standard of hygiene and immune adaptation in newborn infants [15 rehybridized/batch correction samples] (This SubSeries is part of SuperSeries GSE53473: Standard of hygiene and immune adaptation in newborn infants)	Affymetrix	neonatal	WB	XIST	15	GSE53472	21
Genome-wide analysis of gene expression levels in placenta and cord blood samples from newborns babies	Illumina	neonatal	WB, placenta	NA	96	GSE36828	NA
Oxygen induced complication of prematurity: from experimental data to prevention strategy	Affymetrix	neonatal	WB	NA	299	GSE32472	25
Gene expression study reveals compromised Pattern Recognition Receptors and Interferon Signaling in fullterm Low birth Weight newborns	Affymetrix	neonatal	WB	NA	12	GSE29807	NA
Deregulation of Gene Expression induced by Environmental Tobacco Smoke Exposure in Pregnancy	Illumina	neonatal	WB, placenta	NA	104	GSE30032	20
Comprehensive Study of Tobacco Smoke-Related Transcriptome Alterations in Maternal and Fetal Cells	Illumina	neonatal, adult	WB, placenta	NA	183	GSE27272	19
Gene expression profiles of adult peripheral and cord blood mononuclear cells altered by lipopolysaccharide	Affymetrix	neonatal, adult	PBMCs, CBMCs	NA	12	GSE3140	30
The human reticulocyte transcriptome	Affymetrix	fetal, adult	erythroid cells	TFRC	12	GSE17639	31
Expression Profiling of Primary Human Fetal and Adult Hematopoietic Stem/Progenitor Cells (HSPCs) and Differentiating Proerythroblasts (ProeEs); (This SubSeries is part of SuperSeries GSE36994: Comparative profiling of human fetal and adult erythropoiesis)	Affymetrix	fetal, adult	HSCs, erythroid cells	CD34, TFRC	22	GSE36984	32
Expression Profiling of Primary Human Proerythroblasts (ProEs) After IRF2, IRF6, and MYB shRNA Knockdown (This SubSeries is part of SuperSeries GSE36994: Comparative profiling of human fetal and adult erythropoiesis)	Affymetrix	fetal, adult	erythroid cells	TFRC	20	GSE36988	32
Characterization of Transcription Factor Networks Involved in Umbilical Cord Blood CD34+ Stem Cells-Derived Erythropoiesis	Illumina	neonatal	erythroid cells	CD34, TFRC	12	GSE49438	33
Densely interconnected transcriptional circuits control cell states in human hematopoiesis	Affymetrix	neonatal, adult	various cell types	CD34, TFRC, CD19, CD4, CD3D, CD14, CD8A	211	GSE24759	34
Expression data from human CD34+ HPC subpopulations isolated from umbilical cord blood	Affymetrix	neonatal	T/NK and B-lymphoid progenitor cells	CD34, CD19, CD3D	8	GSE29522	NA
Distinct functional programming of human fetal and adult monocytes	Agilent Technologies	fetal, adult	monocytes	CD14	8	GSE54668	35
Restricted Dendritic Cell and Monocyte Progenitors in Human Cord Blood and Bone Marrow	Illumina	neonatal, adult	monocytes, DCs	CD14, CD11C, IL3RA	36	GSE65128	36
DC response to Respiratory syncytial virus from adult peripheral and cord blood	Affymetrix	neonatal, adult	DCs	CD11C, IL3RA	12	GSE24132	37
Differences in the transcriptomic response of human adult and neonatal dendritic cell subsets to TLR7/8 stimulation	Illumina	neonatal, adult	DCs	XIST, IL3RA, CD11C	72	GSE67057	NA
Genome-wide analysis of B lymphocytes derived from human pluripotent stem cells, neonatal and adult sources	Illumina	neonatal, adult	B cells	CD19	19	GSE53572	NA
Functional Analysis and Gene Expression Profile of Umbilical Cord Blood Regulatory T Cells	Affymetrix	neonatal, adult	Tregs	CD4, CD3D, FOXP3	10	GSE22501	NA
Regulatory T cells in human pregnancy	Illumina	fetal, adult	CD4+ T cells	CD4, CD3D	23	GSE31976	38
Comparison of gene expression profiles by CD3+CD4+ thymocytes derived from fetal and adult hematopoietic stem cells (This SubSeries is part of SuperSeries GSE25119: Comparison of CD4+ T cells from human fetal and adult donors)	Affymetrix	fetal, adult	CD4+ T cells	CD4, CD3D, FOXP3	9	GSE25085	39
Human Fetal and Adult Peripheral Naïve CD4+ T cells and CD4+CD25+ Treg cells (This SubSeries is part of SuperSeries GSE25119: Comparison of CD4+ T cells from human fetal and adult donors)	Affymetrix	fetal, adult	CD4+ T cells	CD4, CD3D, FOXP3	12	GSE25087	39
Gene Expression Profile during human CD4+ T cell differentiation (platform GLP96)	Affymetrix	fetal, neonatal, adult	CD4+ T cells	CD4, CD3D	15	GSE1460	40
Gene Expression Profile during human CD4+ T cell differentiation (platform GLP97)	Affymetrix	fetal, neonatal, adult	CD4+ T cells	NA	15	GSE1460	40
Gene expression profile of activated CD4 T cells from adults and newborns	Affymetrix	neonatal, adult	CD4+ T cells	NA	12	GSE52129	41
Expression data from healthy human CD161++CD8aa and CD161++CD8ab T cells (This SubSeries is part of SuperSeries GSE33425: Human MAIT and CD8++ cell development)	Affymetrix	neonatal, adult	CD8+ T cells	CD8A	8	GSE33374	42
Expression data from human cord blood CD161++/ CD161+/CD161-/CD8+ T cell subsets (This SubSeries is part of SuperSeries GSE33425: Human MAIT and CD8++ cell development)	Affymetrix	neonatal, adult	CD8+ T cells	CD8A	9	GSE33424	42

Once a final selection has been made, each dataset was downloaded from GEO using the SOFT file format. For GEO datasets generated using multiple platforms ( GSE1460, GSE25504), the series matrix file format was used instead, and separate datasets for each platform were downloaded. The retrieved datasets were in turn uploaded on an instance of GXB hosted on the Amazon Web Services cloud (39 datasets in total, including 4 SuperSeries and 3 additional datasets that were uploaded due to the use of multiple platforms per GEO dataset). The GXB software has been described in detail in a recent publication ^17^. This custom software interface provides the user with the means to easily navigate and filter the dataset collection, and is available at http://developmentalimmunology.gxbsidra.org/dm3/geneBrowser/list. A web tutorial is also available online: https://gxb.benaroyaresearch.org/dm3/tutorials.gsp#gxbtut. Annotation and functionality of the web software interface were described previously by our group ^27, 28^, and is reproduced here so that readers can use this article as a standalone resource. Available sample and study information were uploaded along with the gene expression data. Samples of each dataset were grouped according to study design and gene rankings were computed for the different group comparisons. Datasets of interest can be quickly identified either by filtering on criteria from pre-defined sections on the left or by entering a query term in the search box at the top of the dataset navigation page. Clicking on one of the studies listed in the dataset navigation page opens a viewer designed to provide interactive browsing and graphic representations of large-scale data in an interpretable format. This interface is designed to present ranked gene lists and display expression results graphically in a context-rich environment. Selecting a gene from the rank ordered list on the left of the data-viewing interface will display its expression values graphically in the screen’s central panel. Directly above the graphical display drop down menus give users the ability: a) To change how the gene list is ranked - this allows the user to change the method used to rank the genes, or to only include genes that are selected for specific biological interest; b) To change sample grouping (Group Set button) - in some datasets, a user can switch between groups based on cell type to groups based on disease type, for example; c) To sort individual samples within a group based on associated categorical or continuous variables (e.g. gender or age); d) To toggle between the bar chart view and a box plot view, with expression values represented as a single point for each sample. Samples are split into the same groups whether displayed as a bar chart or box plot; e) To provide a color legend for the sample groups; f) To select categorical information that is to be overlaid at the bottom of the graph - for example, the user can display gender or smoking status in this manner; g) To provide a color legend for the categorical information overlaid at the bottom of the graph; h) To download the graph as a portable network graphics (png) image. Measurements have no intrinsic utility in absence of contextual information. It is this contextual information that makes the results of a study or experiment interpretable. It is therefore important to capture, integrate and display information that will give users the ability to interpret data and gain new insights from it. We have organized this information under different tabs directly above the graphical display. The tabs can be hidden to make more room for displaying the data plots, or revealed by clicking on the blue “show info panel” button on the top right corner of the display. Information about the gene selected from the list on the left side of the display is available under the “Gene” tab. Information about the study is available under the “Study” tab. Rolling the mouse cursor over a bar chart feature while displaying the “Sample” tab lists any clinical, demographic, or laboratory information available for the selected sample. Finally, the “Downloads” tab allows advanced users to retrieve the original dataset for analysis outside this tool. It also provides all available sample annotation data for use alongside the expression data in third party analysis software. Other functionalities are provided under the “Tools” drop-down menu located in the top right corner of the user interface. Some of the notable functionalities available through this menu include: a) Annotations, which provides access to all the ancillary information about the study, samples and dataset organized across different tabs; b) Cross-project view; which provides the ability for a given gene to browse through all available studies; c) Copy link, which generates a mini-URL encapsulating information about the display settings in use and that can be saved and shared with others (clicking on the envelope icon on the toolbar inserts the URL in an email message via the local email client); d) Chart options; which gives user the option to customize chart labels.

## Quality control

The ‘Copy Link’ function from the “Tools” drop down menu described above was used to generate links to a variety of known hematopoietic markers, allowing the user to perform quality control checks on each dataset by examining the expression profiles of specific sort-purified hematopoietic cell populations, or to determine the degree of contamination of the sample by other cell populations. For our dataset collection, relevant biological indicators included: CD3 (CD3D), a T cell marker; CD4 and CD8 (CD8A), markers of CD4 ^+^ and CD8 ^+^ T cells respectively; FOXP3, a regulatory T cell marker; CD19, a B cell marker; TFRC, a transferrin receptor required for erythropoiesis; CD34, a stem and progenitor cell marker; CD11c (ITGAX), a conventional DC marker; IL-3 receptor alpha (IL3RA), a plasmacytoid DC marker; or CD14, expressed by monocytes and macrophages. For those datasets that contained gender information, we also examined expression of XIST, to determine the concordance between higher XIST expression in female- compared to male samples with the gender information provided with the GEO submission. We hyperlinked this information with the quality control markers given in Table 1 for most of the GEO datasets included in our collection.

## Data availability

The data referenced by this article are under copyright with the following copyright statement: Copyright: © 2016 Rahman M et al.

All datasets included in our curated collection are also available publically via the NCBI GEO website: www.ncbi.gov/geo; and are referenced throughout the manuscript by their GEO accession numbers (e.g. GSE25087). Signal files and sample description files can also be downloaded from the GXB tool under the “downloads” tab.
