# Supplementary material for: A curated transcriptome dataset collection to investigate the functional programming of human hematopoietic cells in early life
Source: F1000Res. 2016 Mar 30;5:414. [Version 1] doi: 10.12688/f1000research.8375.1 (PMC4916988; doi:10.12688/f1000research.8375.1)
Supplement: Peer review history

# Peer review: Referee response for version 1

**DOI:** 10.5256/f1000research.9008.r14443

**Published:** 2016-06-17

**Authors:** Peter Ghazal

**Affiliations:** 1 Division of Infection and Pathway Medicine, Medical School, Edinburgh Infectious Diseases, University of Edinburgh, Edinburgh, UK

The report by Marr and colleagues compiles a valuable set of human early-life publically available expression datasets from the Gene Expression Omnibus (GEO) resource. Obtaining consent and sufficient amounts of sample for this population group is problematical and the limited number of datasets presented reflects the scarcity of studies in this area. The authors have made these datasets web accessible through the Gene Expression Browser (GXB). Interrogating these datasets using GXB application is straightforward but is quite limited providing a restrictive gene analytic view. Incorporating pathway-querying and visualization functions could enhance the overall utility of GXB. Further this would benefit with a note regarding the update frequency for new relevant datasets – during the review process new datasets have already become available. The usefulness and research value of this resource will only be met with continued effort to routinely curate new datasets.

I have read this submission. I believe that I have an appropriate level of expertise to confirm that it is of an acceptable scientific standard.

# Peer review: Referee response for version 1

**DOI:** 10.5256/f1000research.9008.r13168

**Published:** 2016-04-13

**Authors:** Stanislas Goriely

**Affiliations:** 1 WELBIO and Institute for Medical Immunology, Université Libre de Bruxelles, Brussels, Belgium

This report by Nico Marr and colleagues puts together publicly available expression datasets pertinent to the function/development of human immune and hematological cells in early life. The authors use a Web-based application (Gene Expression Browser ^1^) to facilitate exploration and visualization of the data. This tool is useful for the field and user-friendly. However, the current collection is rather heterogenous (clinical samples from septic shock patients, expression profile in progenitor cells after shRNA knockdown for specific transcription factors, general data on different hematopoietic subpopulations without special emphasis on infant/adult comparison...) so it might not be that easy for researchers or clinicians to navigate between the datasets with a specific question in mind. Furthermore, is not clear whether it will be updated on a regular basis. Will other genome-wide datasets (ChIP-Seq, Methylation Arrays..) be incorporated?

I have read this submission. I believe that I have an appropriate level of expertise to confirm that it is of an acceptable scientific standard.

## References

[1] SpeakeCPresnellSDomicoKZeitnerBBjorkAAndersonDMasonMJWhalenEVargasOPopovDRinchaiDJourde-ChicheNChicheLQuinnCChaussabelD: An interactive web application for the dissemination of human systems immunology data. *J Transl Med*.2015;13: 10.1186/s12967-015-0541-x 196 10.1186/s12967-015-0541-x 26088622PMC4474328
